# Chronic air pollution and social deprivation as modifiers of the association between high temperature and daily mortality

**DOI:** 10.1186/1476-069X-13-53

**Published:** 2014-06-18

**Authors:** Tarik Benmarhnia, Youssef Oulhote, Claire Petit, Annabelle Lapostolle, Pierre Chauvin, Denis Zmirou-Navier, Séverine Deguen

**Affiliations:** 1 EHESP School of Public Health, Sorbonne-Paris Cité, Rennes, France; 2 Université de Montréal, DSEST, Montréal, QC, Canada; 3 INSERM U1085 (IRSET), Rennes, France; 4 INSERM UMRS707, DS3, Paris, France; 5 UPMC Univ Paris 06, UMRS 707, Paris, France; 6 Lorraine University Medical School, Vandœuvre-les, Nancy, France

**Keywords:** Heat, Population health, Time-series models, Air pollution, Socioeconomic status

## Abstract

**Background:**

Heat and air pollution are both associated with increases in mortality. However, the interactive effect of temperature and air pollution on mortality remains unsettled. Similarly, the relationship between air pollution, air temperature, and social deprivation has never been explored.

**Methods:**

We used daily mortality data from 2004 to 2009, daily mean temperature variables and relative humidity, for Paris, France. Estimates of chronic exposure to air pollution and social deprivation at a small spatial scale were calculated and split into three strata. We developed a stratified Poisson regression models to assess daily temperature and mortality associations, and tested the heterogeneity of the regression coefficients of the different strata. Deaths due to ambient temperature were calculated from attributable fractions and mortality rates were estimated.

**Results:**

We found that chronic air pollution exposure and social deprivation are effect modifiers of the association between daily temperature and mortality. We found a potential interactive effect between social deprivation and chronic exposure with regards to air pollution in the mortality-temperature relationship.

**Conclusion:**

Our results may have implications in considering chronically polluted areas as vulnerable in heat action plans and in the long-term measures to reduce the burden of heat stress especially in the context of climate change.

## Introduction

Among environmental determinants of health, climatic effects are of high concern, especially given the growing body of literature concerning the impacts of climate change. Ambient temperature has long been recognized as a physical hazard, and is associated with a wide range of adverse health effects [1]. Increases in summer ambient temperatures are associated with increases in mortalities over a city specific threshold. Consequently, future heat-related mortality is likely to increase in the context of climate change [2,3].

Identification of factors that increase vulnerability to hot temperature in human populations has become a growing public health concern [4,5]. Some studies have explored different aspects of heat-related mortality or morbidity heterogeneity across different levels. These heterogeneity factors or effect modifiers in relation to temperature and mortality represent the heat vulnerability factors which can be observed at the individual or at the community level. At the scale of the individual, many studies have reported increased vulnerability of elderly individuals to hot temperatures [6-14]. The effects of gender on temperature impacts have seen mixed evidence [12,13]. At the community level, vulnerability of populations has been shown to be influenced by factors such as low density of green spaces [15], poor urban design and planning [16], urban heat islands [17], and the modification effect by air pollutants [18,19]. Ozone in particular has been studied as an effect modifier in the mortality-temperature relationship [20,21], while the link between temperature and particulate matter (PM) [22], sulfur oxides (SOx) [23] or Nitrogen oxides (NOx) [24] has been less explored. Furthermore, urban air pollution has never been studied from a chronic exposure perspective but only using daily levels. Some studies have also focused on community-based social characteristics that may influence mortality and morbidity outcomes through effects of community organization [25], social cohesion or social networks [26], and social deprivation using an aggregated index [27].

The interaction between ambient temperature and air pollution has been investigated in air pollution mortality time series studies and, to a lesser extent, in temperature mortality time series analyses [28]. To our knowledge, no data are available on the relationship between temperature and mortality effect modified by chronic air pollution exposure and socio-economic factors (or ecological social deprivation) assessed jointly.

The objective of the study is to identify whether and how the magnitude of the effects of mean temperature on all-cause mortality were modified by chronic air pollution exposure (here nitrogen dioxide (NO2) representing urban traffic), social deprivation, and a combination of these two factors. For this purpose, we studied the city of Paris (France) where NO2 long-term average concentrations vary substantially across the city according to traffic density (i.e. the main source of NO2 emissions), and where the different city neighbourhoods host populations with contrasted socio-economic profiles. We hypothesized that a better understanding of these vulnerability factors should provide relevant information for developing public health programs targeting the most vulnerable populations and territories.

## Methods

### Study setting and small-area level

Paris, the capital city of France, has a population of roughly 2.25 million inhabitants. The spatial scale used was the French census block (i.e. IRIS - a French acronym for “blocks for incorporating statistical information”), which constitute the smallest census unit areas in France, designed by the National Census Bureau (INSEE - Institut national de la statistique et des études économiques), for which aggregate data is available. The city of Paris is subdivided into 992 census blocks with a mean population of 2,199 inhabitants (range = 0-5,456 inhabitants) and a mean area of 0.11 km^2^ (range = 0.009-5.4 km^2^).

### Mortality and population data

We considered all deaths occurred in the city of Paris (excluding woods of Boulogne and Vincennes) for residents older than 35 years old from May to August for the years 2004–2009 included. All-cause mortality data were provided by the death registry of the city of Paris. Individual information on age, sex, date of death, and census block of residence was available for each case of death. For confidentiality issues it was not possible to distinguish causes of mortality, thus external causes of deaths could not be excluded. The analysis included only subjects older than 35 years old at the time of death to minimize this bias because accidental causes of death are dominant in subjects under 35 years old [29]. We obtained the number of population in each stratum from the INSEE.

Ethical approval was obtained from the French commission on data privacy and public liberties (CNIL - Commission Nationale Informatique et Liberté).

### *Climate data*

Daily mean outdoor temperatures and relative humidity were obtained from Météo-France, as measured at the Montsouris station in Paris and were computed using data of the corresponding period (summers from 2004 to 2009).

### Air pollution data

Annual N02 concentrations were modeled at a grid scale of 25x25m throughout the period 2004–2009 (only summers) by the local association for the monitoring and the study of air quality (AirParif). We used a dispersion model (ESMERALDA) to produce annual NO2 concentrations at a fine spatial resolution. A description of the methods used to produce NO2 levels at the French census block is provided in Additional file 1. Chronic NO2 exposure at the census block scale was defined as the average of annual NO2 concentrations from 2004 to 2009. We did not include other air pollutants such as ozone due to the lack of data at this spatial scale.

### Community based socio-economic characteristics: social deprivation

To characterize the socio-economic characteristics at a community level, we computed an aggregated, multidimensional deprivation index, fitted at the census block scale [30]. We provided a summary description of the deprivation index and its categorization in Additional file 1.

### *Statistical analyses*

Social deprivation was defined and stratified according to the 3-classes deprivation index described above. NO2 chronic exposure was categorized into three groups according to the terciles of its distribution. To create a double stratification with sufficient statistical power, we stratified the chronic NO2 exposure into two strata according to the median of NO2 chronic concentrations (i.e. we obtained a total of 6 strata for this double stratification). We also assessed age-related vulnerability (i.e. number of daily deaths for the ≥ 65 years people compared to the number of daily deaths for the <65 years) and sex-related vulnerability (number of daily deaths for men versus the number of daily deaths women).

First, in a crude model, we developed stratified Poisson regression models to assess daily temperature-related mortality associations with daily mean temperatures and daily death counts [31]. We used cubic B-splines (5 knots) of time to control for secular trends in the mortality series [9]. Seasonal patterns of mortality were controlled by including a quadratic function represented by the day of the season (1 to 123) [32]. Sensitivity analyses were performed by using a cubic polynomial for seasonality. We used natural cubic splines (2 knots) to consider the non-linearity of the temperature-mortality relationship. Daily levels of humidity were incorporated into regression models as possible confounding variables based on evidence in the literature [1,33,34]. Then, we developed stratified models for each category [35]. The threshold for statistical significance was set at p ≤ 0.05 and all the tests were two-sided. Model assumptions and validity were verified graphically (i.e. with quintile-quintile and partial autocorrelation function plots). A white noise test [32] was also used to ensure that no auto-correlation remained in the residuals. Estimates of exposure (daily temperature) – response (mortality) functions - were expressed as Relative Risks (RR) and their confidence intervals (CI) at 95% for each temperature degree. Relative Risks for the relation between mortality and temperature were estimated relative to the daily mean number of deaths for the entire period [34]. We assessed the heterogeneity of temperature–mortality associations across different strata using the method developed by Payton et al. [36] to compare the regressions’ coefficients between different strata. This test was conducted on all regression coefficients across the strata.

### Calculation of deaths attributable to temperature

We calculated deaths attributable to ambient temperatures for each stratum. Attributable deaths were calculated from attributable fractions (AF), using heat-related mortality relationships (RR) described in the previous section [37,38]. We considered only days with daily mean temperature related to a RR strictly greater than 1 for any temperature value (corresponding to 280 days in total). AF were calculated for each temperature degree (Ti°: represents a temperature unit) and using the equation [(RR(Ti°)-1)/RR(Ti°)].

The Attributable Number of deaths (AN) for the period 2004–2009 (months of May, June, July, and August), for a given temperature variable, was then estimated from the following equation:

$$\mathrm{AN}=\sum_{i=\min}^{\max}\mathrm{AF}\left(T_{i}\right)\times \mathrm{MDC}\times \mathrm{ND}\left(T_{i}\right)$$

In this equation, *i* is the temperature degree Celsius, AF(*T*_*i*_) is attributable fraction for temperature degree (Ti), MDC is the mean observed daily death count, and ND(*T*_*i*_) is the number of days for which temperature was *i* (°C). We then divided the total number of attributable deaths for the period 2004–2009 by 6 to obtain an average attributable number of deaths by summer. We used the RR estimated within the strata of interest to estimate the AR in those strata. We calculated confidence intervals at 95% of attributable number of deaths. Then we compared the number of deaths attributable to temperature in each of the groups stratified by chronic air pollution exposure (3strata), social deprivation (3 strata) and the two simultaneously (6 strata). We made sure that there was no homogeneity in the temperature–mortality associations across different strata, as described in the last section. Finally we calculated attributable mortality rates, by dividing the total number of deaths attributable to temperature in each stratum (by summer) by the total population in each of these strata. The final results were mapped to visualize heat vulnerability according to social deprivation and chronic air pollution exposure.

## Results

**Figure 1:**
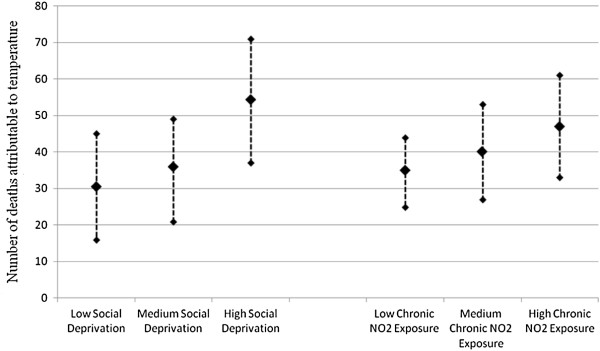
Number of deaths attributable to mean temperature by social deprivation (3 strata) and chronic air pollution exposure (3 strata).

46,056 deaths were registered during the study period, and the mean age at death was 68 years (SD = 10.21). As presented in Table 1, daily death count ranged from 3 to 93 (mean, 33.3, IQR: 27 to 42). Mean temperatures ranged from 7°C to 26°C (mean: 17.3°C, IQR: 14°C to 20°C), while atmospheric pollution assessed by daily NO2 ranged from 16 to 121 μg/m3 (mean: 48.9 μg/m^3^, IQR: 38 μg/m^3^ to 58 μg/m^3^) (Table 1). Chronic NO2 exposure ranged from 39 μg/m^3^ to 81 μg/m^3^ (mean: 52.5 μg/m^3^, IQR: 48 μg/m^3^ to 56 μg/m^3^). From the 992 census blocks in our study, we had 54 census blocks with missing data concerning social deprivation which corresponded to 160 deaths. These census blocks are non-residential (activity and miscellaneous) and with few residents. Descriptive statistics of the social deprivation index and chronic air pollution are presented in (Additional file 1: Table S1). As shown in Additional file 1: Figure S1, chronic NO2 concentrations were slightly higher in the most favoured census blocks. In the crude model (for the whole population), mortality was significantly associated with daily mean temperature (RR for each temperature unit and for all strata are shown in Additional file 1: Table S2 and Table S3). The association between temperature and mortality was U-shaped. Applying the central point estimate of the temperature-mortality relationships to the observed mean temperatures, an average number of 121 attributable deaths by summer (May to August) was estimated. Deaths attributable to mean temperature are presented with their 95% confidence intervals in Additional file 1: Table S4.As presented in the Figure 1, we observed a gradient in the raw deaths attributable to temperature according to social deprivation: the higher the degree of social deprivation, the higher the number of deaths attributable to mean temperature. Likewise, higher chronic air pollution exposure as assessed by NO2 levels was also identified as a vulnerability factor in the relationship between temperature and mortality. We observed a significant difference in regression coefficients (between strata) separately according to social deprivation and chronic air pollution (p < 0.05).

**Table 1:** Summary statistics for health outcomes, climate and air pollutants data (Paris, 2004–2009)

**Variable**	**Mean**	**Minimum**	**25th Percentile**	**Median**	**75th Percentile**	**Maximum**	**Standard deviation**
Daily death count (n)	33.3	3	27	33	42	93	4.5
Minimum Temperature (°C)*	12.5	0	9	13	16	21	4.2
Mean Temperature (°C)*	17.3	7	14	17	20	26	4.4
Maximum Temperature (°C)*	20.9	8	17	21	24	35	5.2
Relative Humidity (%)*	68.4	37	61	69	76	94	10.7
Daily NO2 (μg/m^3^)	48.9	16	38	47	58	121	14.3
Chronic NO2 (μg/m^3^)	52.5	38.7	47.88	51.8	56.1	81.1	6.8

*Summary statistics for weather variables (minimum, mean and maximum temperatures as well as relative humidity) are based on daily data.

Finally, we calculated mortality rates (Table 2), including the population living in each strata to consider the differences in population density across strata. We found some heterogeneity (p < 0.10) between ambient temperature and mortality according to age, social deprivation and chronic air pollution exposure. Sex was not an effect modifier in the relationship between heat and temperature (p = 0.51). For the double stratification, we found that, in the low chronic exposure group, social deprivation did not significantly modify the relation between heat and mortality (p = 0.14), while we found that social deprivation may have modified this relation in the low chronic exposure group (p = 0.07). We also conducted heterogeneity tests across the two chronic exposure groups. We found a heterogenic effect (data not shown) only between the strata of low chronic exposure/high social deprivation and the strata of high chronic exposure/ high social deprivation.

**Table 2:** Mortality rates attributable to summer temperatures (per 100 000) by strata

***Strata***	***Population***	***Mortality rates (per 100 000)***^***a***^	***p for heterogeneity***^***b***^
**Total**	2234105	5.37 [5.01;5.73]	
**Age**			
Under 65 years	1921330	0.78 [0.62;0.88]	0.001
More than 65 years	312774	33.57 [31.65;35. 81]	
**Sex**			
Female	1161734	5.51 [4.99;5.85]	0.51
Male	1072370	5.22 [4.76;5.67]	
**Social Deprivation**			
Low Social Deprivation	692572	4.33 [3.31;5.23]	0.08
Medium Social Deprivation	781936	4.60 [3.59;5.41]	
High Social Deprivation	743956	7.26 [6.74;7.85]	
**Chronic air pollution exposure**			
Low Chronic NO2 Exposure	737254	4.75 [4.13;5.22]	0.03
≤ 50.6 μg/m^3^			
Medium Chronic NO2 Exposure	795341	5.97 [5.36;6.32]	
50.6-55.8 μg/m^3^			
High Chronic NO2 Exposure	670231	7.89 [7.28;8.14]	
> 55.8 μg/m^3^			
**Double stratification Low Chronic Exposure Group**			
Low Social Deprivation	370567	3.78 [2.87;5.03]	0.14
Medium Social Deprivation	381190	4.19 [3.34;5.25]	
High Social Deprivation	375341	6.92 [5.11;8.12]	
**Double stratification High Chronic Exposure Group**			
Low Social Deprivation	389872	3.59 [2.29;5.07]	0.07
Medium Social Deprivation	391432	5.36 [4.22;6.41]	
High Social Deprivation	325701	9.82 [7.79;10.93]	

^a^For social deprivation and chronic air pollution strata, mortality rates were standardized for age and sex.

^b^Homogeneity test of Chi- square Pearson, for central estimates.

**Figure 2:**
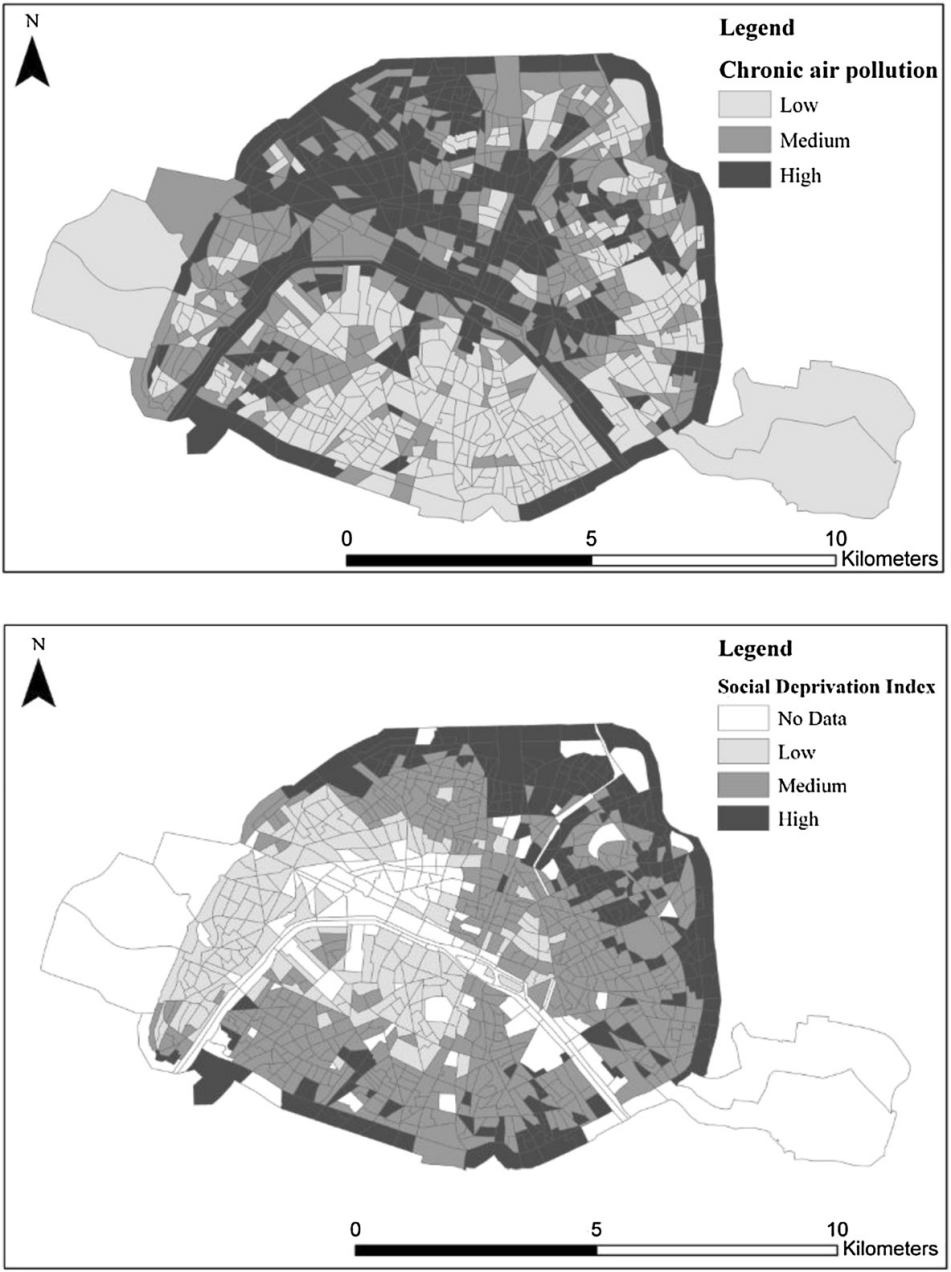
Spatial distribution of social deprivation and chronic air pollution.

Figure 2 presents the spatial distribution of social deprivation and chronic air pollution exposure. The spatial variations of both social characteristics are related to deaths attributable to temperature as presented in Table 2.

## Discussion

In this study, we assessed the effects of mean temperature and chronic air pollution (with NO2 representing urban traffic) on all-cause mortality with additional attention paid to the interactive effects of social deprivation. To do this we conducted stratified time-series analyses. We found that chronic air pollution exposure modifies the association between daily temperature and mortality. We also found that social deprivation is a heat-related vulnerability factor. Finally we found that there is a potential combined modification effect of social deprivation and chronic exposure to NO2 with regards to heat-related mortality.

Our results for the temperature-mortality relationship are comparable to other studies conducted in Paris [8,32]. Concerning social vulnerability to heat-related mortality, our results are consistent with other studies, using time series analyses [5] or using different methods, such as a case crossover design [11].

Ecological social vulnerability can be explained by an accumulation of different types of vulnerabilities for individuals within a census block (e.g., social isolation [39], material conditions [40], poor urban design including micro heat islands [17] or poorer health [14]). In the same way, social mobility may also partly explain the observed social gradient, similar to previous studies looking at socio-economic scales [41]. Thus, populations with low socio-economic levels will be inclined to stay in their neighbourhoods, with a low daily mobility, accumulating other heat vulnerability factors and with other consequences on certain determinants of health such as physical activity.

We found that air pollution modifies the relation between ambient temperature and daily mortality as found in previous studies using daily exposure [21-23,28,42]. It is possible that chronic NO2 levels modify the effects of temperature on mortality. A range of studies have shown that NO2 is consistently associated with many health outcomes such as cardiovascular effects [43,44], which can explain how the NO2 chronic effects can make some populations more vulnerable to heat impacts.

Our results suggest that ambient temperature has a greater impact on daily mortality on chronically polluted areas, especially if these areas are socially deprived. However our results only suggest this double effect modifier and further studies may confirm this result.

Methods for estimating the heat related mortality relationships inevitably rely on some assumptions. In our study, we chose to not take into account lag effects to simplify the interpretation of deaths attributable to temperature. We considered that contribution of lag effects compared to the day of death was not differential according to the different strata characteristics. We did not take into account harvesting effect or mortality displacement. We also did not consider intra urban variations of temperatures. Future studies should consider these aspects. We considered the social deprivation at the ecological level, and we did not explored social inequalities in mortality at the individual level, because of the lack of data. The use of a synthetic index rather than using independent ecological socio-economic measures allows us to represent an accumulation of social and material disadvantages [45] and these kinds of indexes are more useful to lead population interventions [27]. The division of neighbourhoods into census blocks aims to maximize their homogeneity in terms of population size, socioeconomic characteristics, land use and zoning. In Paris, the area of a block census is quite small so the ecologic bias is likely to be small. Finally, we did not consider the spatial auto-correlation in our analysis which is a result of the correlation between the spatial unit and the adjacent geographical areas. Variables observed at small area level are interdependent because proximity and linkages between neighbouring areas. This issue could influence the effect of social deprivation and chronic air pollution [46], and further studies could consider it by conducting spatial analyses such as a hierarchical Bayesian modeling approach.

## Conclusion

In this study, we showed that areas which are chronically polluted by air pollution can be characterized as vulnerable to heat in the sense that they have more deaths attributable to heat than less exposed areas. We also presented a potential combined vulnerability of social deprivation and chronic exposure to air pollution in the mortality-temperature relationship. Our results may have important implications considering chronically polluted areas as vulnerable in heat actions plans (especially including adapted surveillance and warning systems) and in the long-term measures to reduce the burden of heat stress (as building regulations, urban planning or land-use changes), especially in the context of climate change. However, further studies are necessary to determine whether similar results could be found in other settings.

## Ethics approval

French commission on data privacy and public liberties (CNIL - Commission Nationale Informatique et Liberté).

## Abbreviations

AF: Attributable fraction; CI: Confidence interval; INSEE: Institut National de la Statistique et des Etudes Economiques (National Census Bureau); IQR: Inter quartile range; NO: Nitrogen oxides; PM: Particle matter; RR: Relative risk; SD: Standard deviation; SES: Socio economic status; SO: Sulfur oxides.

## Competing interests

The authors declare that they have no competing interests.

## Authors’ contributions

TB, YO and SD conceived the study. TB and CP analyzed the data in consultation with SD and DZN. TB, CP and YO wrote the draft version and revisions of the manuscript. SD, DZN, AL and PC contributed to the writing of the manuscript. All authors agree with manuscript results and conclusions. All authors read and approved the final version of the manuscript.

## Supplementary Material

Additional file 1 Supplemental Material. Click here for file
