# Supplementary material for: Chronic air pollution and social deprivation as modifiers of the association between high temperature and daily mortality
Source: Environ Health. 2014 Jun 18;13:53. doi: 10.1186/1476-069X-13-53 (PMC4073194; doi:10.1186/1476-069X-13-53)
Supplement: Additional file 1 — Supplemental Material. [file 1476-069X-13-53-S1.docx]

**Supplemental Material**

**Description of the methods used to produce daily NO2 levels**

First, annual NO2 concentrations were modelled from a grid of 25x25m resolution throughout the period 2002–2009 by the local association for the monitoring and the study of air quality (AirParif: http://www.airparif.asso.fr/). They used the ESMERALDA dispersion model (ESMERALDA 2012) for background pollution and the STREET dispersion model (Oxalys Scop SA, Broissieux, France) for pollution related to traffic proximity. To compute annual NO2 concentrations at a fine spatial resolution (25x25m), these models incorporated several types of input data: emission inventories, meteorological data and background pollution measurements, supplied respectively by industry and environment regional administration, Météo-France (French meteorological agency) and monitoring stations of the regional network. Air pollutant concentrations were then aggregated at the census block scale in order to obtain the annual mean of NO2 concentration for each census block. The aggregation technique was a population-weighted average.

**Summary description of the Deprivation Index**

To characterize the socioeconomic status, we used an index developed at the census block scale for Paris. Briefly, a principal component analysis was used to select variables among 41 socioeconomic and demographic variables provided by the 2006 national census at the census block scale. Following the results of this principal component analysis, 15 variables were best correlated (based on the contribution) with the first component: median income, percentage of people with basic or intermediate general or vocational qualifications, people with a higher educational degree, unemployed, self-employed, non-graduates, non-owners, housing with floor area more than 100 m², subsidized housings, foreign immigrants, artisans, managers, employees, blue-collar workers, and single-parent families. These 15 variables were selected to carry out a final principal component analysis where the reduced first component was used to calculate the socioeconomic index. Finally, an ascendant hierarchical analysis was performed to gather census blocks in 3 homogenous socioeconomic categories numbered from 1 (the most privileged) to 3 (the most deprived). About 13% (n = 126) of census blocks were not classified in a socioeconomic category because they corresponded to non-residential census blocks (activity and miscellaneous) with few residents. Category 1 is characterized by census blocks with high median incomes and high percentages of housings with area greater than 100 m², self-employed, artisans, managers, and people with a higher educational degree. Only two variables positively characterized the census blocks of category 2: percentage of managers and people with a higher educational degree are over-represented, while the census blocks with the other variables are under-represented. The most deprived category (category 3) is represented by a high percentage of non-graduates, blue-collar workers, employees, subsidized housings, single-parent families, unemployed, people with basic or intermediate general or vocational qualifications, non-owners, and foreign immigrants.

**Calculation of the 95% limits of summer deaths attributable to temperature.**

$$LowerCI AN=LCI \overline{{AF}_{Day}}\times MDC \times123$$

$$UpperCI AN=UCI \overline{{AF}_{Day}}\times MDC \times123$$

Where AN represents the summer deaths attributable to temperature, MDC the mean daily deathcount, and 123 the number of days in May, June, July and August. $\overline{{AF}_{Day}}$ is the mean AF (RR-1/RR) per day calculated averaged by number of days for each temperature unit.

CI $\overline{{AF}}$ is obtained from the following equation:

CI95% ln (1 -$\overline{{AF}}$) = ln (1 -$\overline{{AF}}$) $\pm$ 1.96 $\times$ $\sqrt{\overline{Var}}$

And $\overline{VAR}= \frac{\sum_{i=min T^{\circ}}^{\max T^{\circ}} VAR \left( T^{\circ} \right)\times nb of days (T^{\circ})}{\sum_{i=\min T^{\circ}}^{max T^{\circ}} nb of days (T^{\circ})}$

Where VAR = (Standard error of regression estimate)^2^

Consequently,

LCI $\overline{{AF}}$ = 1 – exp [LCI $\overline{{AF}}]$

UCI $\overline{{AF}}$ = 1 – exp [UCI $\overline{{AF}}]$

**Table 1S:** Summary statistics for Social Deprivation and Chronic Air Pollution (Paris, 2004-2009)

| Variable |  | Mean | Minimum |  | 25th  Percentile | Median | 75th Percentile |  | Maximum | Standard Deviation |  |
| --- | --- | --- | --- | --- | --- | --- | --- | --- | --- | --- | --- |
| Social Deprivation | -0.06 | | -6.25 | | -2.39 | -0.81 | 1.62 | 9.70 | | 3.05 |  |
|  |  | |  | |  |  |  |  | |  |  |
| Chronic NO2 (µg/m^3^) |  | 52.5 | 38.7 |  | 47.88 | 51.8 | 56.1 |  | 81.1 | 6.8 |  |

**Figure 1S: Distribution of NO2 levels by quintile of social deprivation**


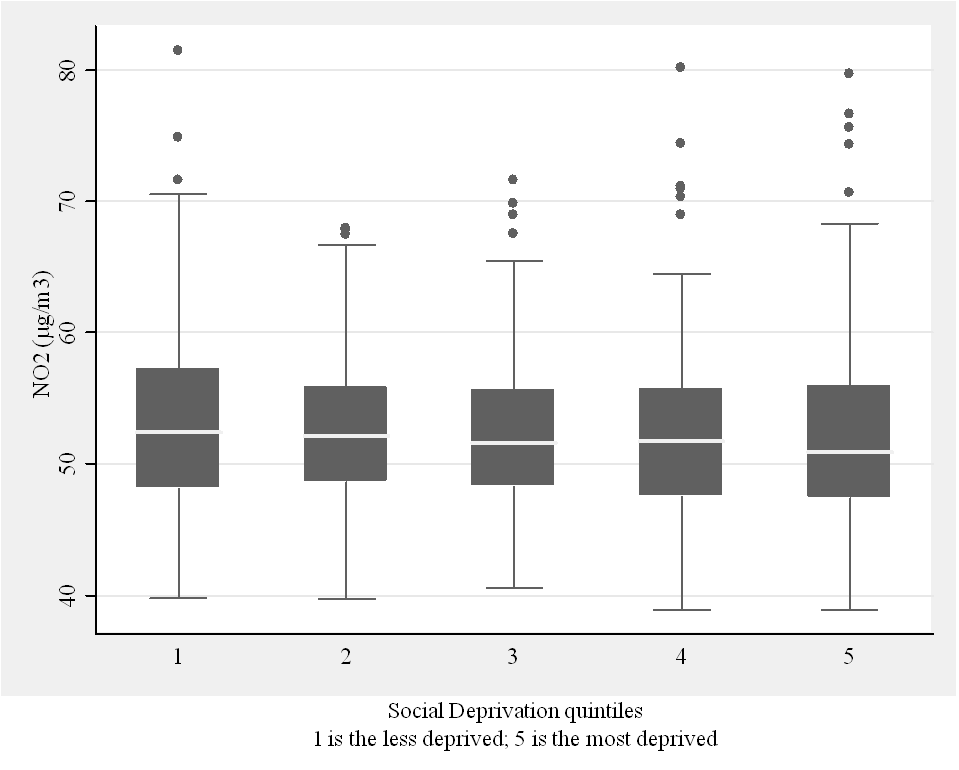


**Table 2S:** RR estimates for Social Deprivation strata

|  | **Low Social Deprivation** | | | **Medium Social Deprivation** | | | **High Social Deprivation** | | |
| --- | --- | --- | --- | --- | --- | --- | --- | --- | --- |
| Mean Temperature (°C) | RR | LCI | UCI | RR | LCI | UCI | RR | LCI | UCI |
| 7 | 1.020 | 0.895 | 1.162 | 1.024 | 0.900 | 1.165 | 1.027 | 0.903 | 1.168 |
| 8 | 1.007 | 0.899 | 1.128 | 1.011 | 0.904 | 1.131 | 1.014 | 0.907 | 1.134 |
| 9 | 0.994 | 0.903 | 1.095 | 0.998 | 0.908 | 1.098 | 1.001 | 0.910 | 1.102 |
| 10 | 0.982 | 0.906 | 1.064 | 0.986 | 0.911 | 1.067 | 0.989 | 0.914 | 1.070 |
| 11 | 0.970 | 0.910 | 1.034 | 0.974 | 0.914 | 1.038 | 0.977 | 0.917 | 1.041 |
| 12 | 0.959 | 0.913 | 1.007 | 0.963 | 0.917 | 1.011 | 0.966 | 0.920 | 1.014 |
| 13 | 0.949 | 0.915 | 0.986 | 0.954 | 0.919 | 0.990 | 0.957 | 0.922 | 0.993 |
| 14 | 0.942 | 0.916 | 0.970 | 0.947 | 0.921 | 0.974 | 0.950 | 0.923 | 0.977 |
| 15 | 0.938 | 0.916 | 0.960 | 0.945 | 0.924 | 0.968 | 0.948 | 0.926 | 0.971 |
| 16 | 0.937 | 0.917 | 0.957 | 0.944 | 0.924 | 0.965 | 0.947 | 0.927 | 0.968 |
| 17 | 0.940 | 0.921 | 0.959 | 0.947 | 0.928 | 0.967 | 0.950 | 0.931 | 0.969 |
| 18 | 0.947 | 0.930 | 0.965 | 0.955 | 0.938 | 0.972 | 0.973 | 0.956 | 0.990 |
| 19 | 0.960 | 0.944 | 0.976 | 0.967 | 0.952 | 0.983 | 1.000 | 0.985 | 1.016 |
| 20 | 0.975 | 0.958 | 0.993 | 0.992 | 0.975 | 1.010 | 1.025 | 1.008 | 1.043 |
| 21 | 0.988 | 0.971 | 1.006 | 1.005 | 0.988 | 1.023 | 1.038 | 1.021 | 1.056 |
| 22 | 1.009 | 1.001 | 1.017 | 1.017 | 1.006 | 1.031 | 1.050 | 1.034 | 1.067 |
| 23 | 1.016 | 1.004 | 1.029 | 1.033 | 1.015 | 1.052 | 1.067 | 1.048 | 1.085 |
| 24 | 1.041 | 1.024 | 1.058 | 1.058 | 1.038 | 1.074 | 1.091 | 1.071 | 1.112 |
| 25 | 1.074 | 1.053 | 1.096 | 1.091 | 1.070 | 1.112 | 1.124 | 1.103 | 1.145 |
| 26 | 1.114 | 1.088 | 1.141 | 1.130 | 1.104 | 1.157 | 1.164 | 1.137 | 1.190 |

LCI: Lower Confidence Intervals; UCI: Upper Confidence Intervals**Table 3S:** RR estimates for Chronic NO2 exposure strata

|  | **Low NO2 exposure** | | | **Medium NO2 exposure** | | | **High NO2 exposure** | | |
| --- | --- | --- | --- | --- | --- | --- | --- | --- | --- |
| Mean Temperature (°C) | RR | LCI | UCI | RR | LCI | UCI | RR | LCI | UCI |
| 7 | 1.047 | 0.922 | 1.188 | 1.050 | 0.925 | 1.192 | 1.058 | 0.933 | 1.201 |
| 8 | 1.034 | 0.926 | 1.155 | 1.037 | 0.929 | 1.158 | 1.045 | 0.937 | 1.167 |
| 9 | 1.021 | 0.930 | 1.122 | 1.024 | 0.933 | 1.125 | 1.033 | 0.941 | 1.134 |
| 10 | 1.009 | 0.934 | 1.090 | 1.012 | 0.937 | 1.094 | 1.020 | 0.944 | 1.102 |
| 11 | 0.997 | 0.937 | 1.061 | 1.000 | 0.940 | 1.064 | 1.008 | 0.947 | 1.073 |
| 12 | 0.996 | 0.950 | 1.044 | 0.990 | 0.953 | 1.048 | 1.007 | 0.960 | 1.056 |
| 13 | 0.987 | 0.952 | 1.023 | 0.974 | 0.955 | 1.022 | 0.998 | 0.963 | 1.034 |
| 14 | 0.979 | 0.953 | 1.007 | 0.973 | 0.954 | 1.021 | 0.990 | 0.964 | 1.018 |
| 15 | 0.975 | 0.953 | 0.998 | 0.978 | 0.958 | 1.024 | 0.987 | 0.965 | 1.010 |
| 16 | 0.974 | 0.954 | 0.995 | 0.977 | 0.957 | 0.998 | 0.986 | 0.966 | 1.007 |
| 17 | 0.977 | 0.958 | 0.996 | 0.980 | 0.961 | 0.999 | 0.989 | 0.970 | 1.008 |
| 18 | 0.980 | 0.937 | 1.002 | 0.982 | 0.970 | 1.005 | 0.991 | 0.979 | 1.014 |
| 19 | 0.983 | 0.951 | 1.013 | 0.985 | 0.994 | 1.026 | 1.002 | 1.000 | 1.035 |
| 20 | 0.987 | 0.957 | 1.030 | 1.009 | 1.001 | 1.014 | 1.018 | 1.003 | 1.029 |
| 21 | 1.000 | 0.991 | 1.043 | 1.022 | 1.011 | 1.030 | 1.031 | 1.020 | 1.042 |
| 22 | 1.012 | 1.001 | 1.053 | 1.034 | 1.021 | 1.047 | 1.043 | 1.032 | 1.056 |
| 23 | 1.028 | 1.015 | 1.072 | 1.051 | 1.039 | 1.067 | 1.060 | 1.047 | 1.081 |
| 24 | 1.053 | 1.038 | 1.099 | 1.075 | 1.059 | 1.097 | 1.085 | 1.069 | 1.105 |
| 25 | 1.086 | 1.062 | 1.122 | 1.108 | 1.078 | 1.135 | 1.118 | 1.098 | 1.132 |
| 26 | 1.126 | 1.096 | 1.177 | 1.148 | 1.111 | 1.200 | 1.158 | 1.115 | 1.211 |

LCI: Lower Confidence Intervals; UCI: Upper Confidence Intervals

**Table 4S:** Summer deaths attributable to mean temperature presented with their 95% CI.

|  |  | Deaths attributable to T° and percentage attributable to temperature* | LCI | UCI |
| --- | --- | --- | --- | --- |
| Social Deprivation | Low Social Deprivation | 30 (9%) | 16 | 45 |
|  | Medium Social Deprivation | 36 (10%) | 21 | 49 |
|  | High Social Deprivation | 54 (14%) | 37 | 71 |
|  |  |  |  |  |
| Chronic Air Pollution | Low Chronic NO2 Exposure | 35 (10%) | 25 | 44 |
|  | Medium Chronic NO2 Exposure | 40 (11%) | 27 | 53 |
|  | High Chronic NO2 Exposure | 47 (13%) | 33 | 61 |
|  |  |  |  |  |
| **Double Stratification** | | | | |
| Low Chronic NO2 Exposure | Low Social Deprivation | 13 (9%) | 4 | 21 |
|  | Medium Social Deprivation | 17 (11%) | 9 | 27 |
|  | High Social Deprivation | 26 (13%) | 17 | 34 |
|  |  |  |  |  |
| High Chronic NO2 Exposure | Low Social Deprivation | 14 (9%) | 6 | 23 |
|  | Medium Social Deprivation | 20 (12%) | 12 | 29 |
|  | High Social Deprivation | 32 (14%) | 23 | 39 |

T°: Temperature

LCI: Lower Confidence Interval

UCI: Upper Confidence Interval

*: percentages attributable to temperature are rounded up to the whole number and are obtained from the average attributable fraction by strata weighted by the number of days by temperature value.

**Figure 2S:** Descriptive map for mortality rates in Paris by census block

**
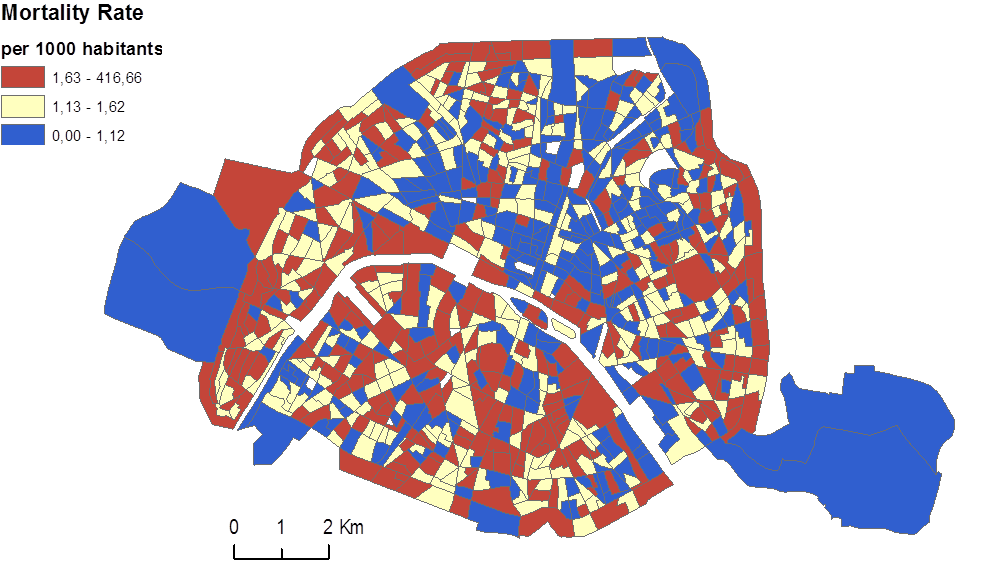
**

**Figure 3S:** Daily time series plot for the death in Paris.

**Figure 4S:** Daily time series plot for the mean temperature in Paris.

**Figure 5S:** Daily time series plot for the NO2 in Paris.
